# Risk Factors Associated With Extended Length of Hospital Stay After Geriatric Hip Fracture

**DOI:** 10.5435/JAAOSGlobal-D-21-00073

**Published:** 2021-05-04

**Authors:** Andrew M. Schneider, Steven Denyer, Nicholas M. Brown

**Affiliations:** From the Department of Orthopaedic Surgery and Rehabilitation, Loyola University Medical Center, Maywood, IL.

## Abstract

**Introduction:**

Within the geriatric hip fracture population, there exists a subset of patients whose length of inpatient hospital stay is excessive relative to the average. A better understanding of the risk factors associated with this group would be of value so that targeted prevention efforts can be properly directed. The goal of this study was to identify and characterize the risk factors associated with an extended length of hospital stay (eLOS) in the geriatric hip fracture population. In addition, a statistical model was created to predict the probability of eLOS in a geriatric hip fracture patient.

**Methods:**

The National Surgical Quality Improvement Program database (2005 to 2018) was searched for patients aged ≥65 years who underwent hip fracture surgery. Patients with a hospital stay greater than or equal to 14 days were considered to have an eLOS. A multivariate logistic regression model using 24 patient characteristics from two-thirds of the study population was created to determine independent risk factors predictive of having an eLOS; the remaining one-third of the population was used for internal model validation. Regression analyses were performed to determine preoperative and postoperative risk factors for having an eLOS.

**Results:**

A total of 77,144 patients were included in the study. Preoperatively, male sex, dyspnea, ventilator use, chronic obstructive pulmonary disease, American Society of Anesthesiologist class 3 and 4, and increased admission-to-operation time were among the factors associated with higher odds of having an eLOS (all *P* < 0.001). Postoperatively, patients with acute renal failure had the highest likelihood of eLOS (odds ratio [OR] 7.664), followed by ventilator use >48 hours (OR 4.784) and pneumonia (OR 4.332).

**Discussion:**

Among geriatric hip fracture patients, particular efforts should be directed toward optimizing those with preoperative risk factors for eLOS. Preemptive measures to target the postoperative complications with the strongest eLOS association may be beneficial for both the patient and the healthcare system as a whole.

In today's cost-conscious medical climate, optimizing healthcare resource utilization among all patients is a worthwhile goal, regardless of specialty. However, there are recent data to suggest that understanding and targeting the costliest users might be an especially impactful strategy, as there exists a subset of patients responsible for a disproportionately large amount of healthcare dollars. For example, in a given year, just 1% of the United States population is responsible for 28% of total healthcare spending, and 5% accounts for over 50 percent.^1,2^ In 2012, if one patient in the top 5% of annual heart disease spending was instead an average user, approximately $59,000 would have been saved.^3,4^ Characterizing the risk factors associated with this population is challenging, but recently, some attempts have been made. Gil et al^5^ compared trauma patients with the longest hospital stays (top 0.06%) with all other trauma patients and found that those with the longest length of stay (LOS) had higher rates of almost every inpatient complication; the strongest associations included pneumonia, decubitus ulcer, acute respiratory distress syndrome (ARDS), deep vein thrombosis, severe sepsis, and acute kidney injury (AKI). In a similar trauma population, Lagoe et al^6^ found that patients who developed pneumonia stayed an average of 12.7 days longer in the hospital than those who did not. These findings suggest that these patients, regardless of their baseline medical condition, may not have been destined for an extended inpatient hospital stay on admission, and the active pursuit of prevention of these risk factors responsible for an extended length of stay (eLOS) could have a profound effect on overall resource use.

The treatment of hip fractures in the elderly has an enormous effect on the economic burden of the United States healthcare system, with an estimated cost of $17 to $20 billion in 2010 alone, as these injuries almost universally require hospitalization and surgical treatment.^7,8^ Although implementations such as orthogeriatric comanagement programs have been shown to decrease LOS and lower inpatient hospitalization cost, geriatric hip fractures still continue to be a disproportionately large resource consumer: hip fractures represent only 14% of geriatric fractures, yet they account for approximately 72% of the total costs of care.^9,10,11,12^ Garcia et al^9^ found the American Society of Anesthesiologist's (ASA) classification, a measure of the sum of a patient's comorbidities, to have the strongest association with increased LOS in this population. Each increase in ASA classification added an extra $9300 to the daily hospital stay.

Identifying the risk factors unique to the geriatric hip fracture patients with the longest hospital stays is paramount to understanding the significant drivers of resource utilization in this population. The objective of this study was to identify and characterize the demographic and clinical factors associated with eLOS after a geriatric hip fracture. In addition, we sought to create a predictive model for eLOS using a large national database.

## Methods

**Figure 1:**
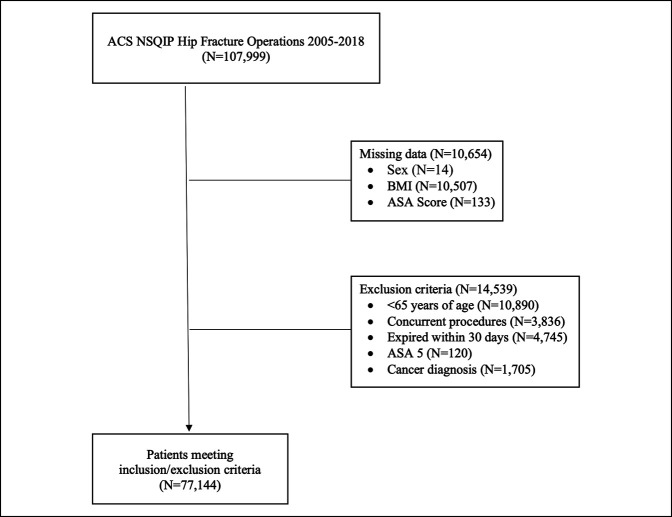
Flowchart demonstrating the formation of the final study population generated from the National Surgical Quality Improvement Program database.

A retrospective review of data collected from the National Surgical Quality Improvement Program (NSQIP) database was performed for the years 2005 to 2018. Primary Current Procedural Terminology codes 27125, 27130, 27235, 27236, 27244, and 27245 were investigated as the hip fracture procedures of interest. Selection was limited to hip fracture diagnoses only, coded using the *International Classification of Diseases Ninth and Tenth* diagnostic codes (Supplemental Digital Content 1, Table 1, http://links.lww.com/JG9/A127). A detailed summary of patient selection and exclusion can be found in Figure 1. This study was exempt from institutional review board approval due to the deidentified nature of the databases, and no funding sources were used.

Demographic data analyzed included sex, race, age, functional status, obesity, and ASA classification. Body mass index was calculated using the National Institutes of Health conversion formula. Comorbidities recorded were diabetes, hypertension, chronic obstructive pulmonary disease (COPD), bleeding disorders, ventilator use, ascites, congestive heart failure, renal failure, dialysis, preoperative sepsis, and smoking status. Transfer status and admission-to-surgery time were also included in our analysis. Patients with a LOS ≥14 days were categorized as having an eLOS. This length of time was chosen based on clinical judgment to reflect a small group of outliers with an unexpectedly long hospital stay. A LOS of 14 days or more corresponded to the top 5% of all patients included in the analysis.

A stepwise logistic regression model predictive for having an eLOS was created using the aforementioned patient variables. The model was built from a random selection of two-thirds of the study's NSQIP population; the remaining one-third was used for model validation.

Finally, additional analyses were performed to assess for differences in preoperative and postoperative complications between eLOS and non-eLOS patients.

Population variables from the NSQIP database were summarized with descriptive statistics. A multivariate logistic regression was used to determine which preoperative characteristics were associated with eLOS. Stepwise methods of selection were applied to find the best set of significant predictors. This method of regression model building relies on statistical criteria for variable inclusion in the model. This method was chosen because although some studies have demonstrated risk factors for complications, it was unknown which clinical factors would be associated with long lengths of stay (>14 days). Therefore, a large pool of potentially clinically relevant variables was initially chosen, and stepwise regression was used to find the most predictive variables. The likelihood ratio test statistic *P* values for entry and removal in the model were set to 0.1. However, because stepwise regression functionally tests multiple hypotheses, our *P* values for statistical significance were adjusted using the Bonferroni correction. The fit of the final reduced model was assessed using the Hosmer-Lemeshow test (*P* = 0.3287). The strength of prediction was evaluated with analysis of the area under the receiver operating characteristic curve (AUC) and Brier score. A univariate analysis and a multivariate logistic regression were used to determine which postoperative characteristics were associated with eLOS. Analyses were performed using SAS software (SAS Institute).

## Results

A total of 77,144 geriatric hip fracture patients from the NSQIP database met the inclusion criteria. The population consisted predominantly of women (n = 55,481; 71.9%) and had a mean age of 82 (± 7.3) years. Additional population characteristics are reported in Table 1.

**Table 1:** Baseline Characteristics

Variable	Total		Non-eLOS		eLOS		*P*
	N	%	N	%	N	%	
Total patients	77,144	100	73,232	100	3912	100	
Surgery							**<0.0001**
Arthroplasty	3833	5	3717	5.1	116	3	** **
Hemiarthroplasty	11,603	15	10,881	14.9	722	18.5	
Internal fixation	61,708	80	58,634	80.1	3074	78.6	
Sex							**<0.0001**
Female	55,481	71.9	53,060	72.5	2421	61.9	
Male	21,663	28.1	20,172	27.5	1491	38.1	
Age							**0.0549**
65-74 yr	14,196	18.4	13,485	18.4	711	18.2	
75-84 yr	26,878	34.8	25,576	34.9	1302	33.3	
85+ yr	36,070	46.8	34,171	46.7	1899	48.5	
BMI							**0.0003**
Normal	37,782	49	35,888	49	1894	48.4	
Obese I	7596	9.8	7197	9.8	399	10.2	
Obese II	2135	2.8	2018	2.8	117	3	
Obese III	1039	1.3	968	1.3	71	1.8	
Overweight	21,900	28.4	20,864	28.5	1036	26.5	
Underweight	6692	8.7	6297	8.6	395	10.1	
Race							**<0.0001**
Unknown	9566	12.4	7681	10.5	1885	48.2	
Black	2474	3.2	2345	3.2	129	3.3	
White	62,809	81.4	61,026	83.3	1783	45.6	
Other	2295	3	2180	3	115	2.9	
Transfer							**<0.0001**
Unknown	4815	6.2	4552	6.2	263	6.7	
Care facility	7619	9.9	7172	9.8	447	11.4	
OSH	5848	7.6	5606	7.7	242	6.2	
Home	58,862	76.3	55,902	76.3	2960	75.7	
Diabetes							**<0.0001**
No	63,286	82	60,186	82.2	3100	79.2	
Yes	13,858	18	13,046	17.8	812	20.8	
Hx smoking							**0.0004**
No	69,868	90.6	66,388	90.7	3480	89	
Yes	7276	9.4	6844	9.3	432	11	
Dyspnea							**<0.0001**
Unknown	1	0	1	0	.	.	
At rest	821	1.1	731	1	90	2.3	
Moderate exertion	4626	6	4240	5.8	386	9.9	
None	71,696	92.9	68,260	93.2	3436	87.8	
Ventilator dependent							**<0.0001**
No	77,035	99.9	73,161	99.9	3874	99	
Yes	109	0.1	71	0.1	38	1	
History of severe COPD							**<0.0001**
No	68,772	89.1	65,519	89.5	3253	83.2	
Yes	8372	10.9	7713	10.5	659	16.8	
Ascites							**<0.0001**
No	77,034	99.9	73,147	99.9	3887	99.4	
Yes	110	0.1	85	0.1	25	0.6	
CHF							**<0.0001**
No	74,541	96.6	70,914	96.8	3627	92.7	
Yes	2603	3.4	2318	3.2	285	7.3	
HTN							0.2155
No	23,302	30.2	22,155	30.3	1147	29.3	
Yes	53,842	69.8	51,077	69.7	2765	70.7	
Acute renal failure							**<0.0001**
No	76,739	99.5	72,889	99.5	3850	98.4	
Yes	405	0.5	343	0.5	62	1.6	
Dialysis (preoperative)							**<0.0001**
No	75,891	98.4	72,131	98.5	3760	96.1	
Yes	1253	1.6	1101	1.5	152	3.9	
Open wound/wound infection							**<0.0001**
No	74,169	96.1	70,507	96.3	3662	93.6	
Yes	2975	3.9	2725	3.7	250	6.4	
Steroid use for chronic condition							**0.0168**
No	73,476	95.2	69,781	95.3	3695	94.5	
Yes	3668	4.8	3451	4.7	217	5.5	
Bleeding disorders							**<0.0001**
No	64,205	83.2	61,094	83.4	3111	79.5	
Yes	12,939	16.8	12,138	16.6	801	20.5	
Transfusion (preoperative)							**<0.0001**
No	74,078	96	70,472	96.2	3606	92.2	
Yes	3066	4	2760	3.8	306	7.8	
Preoperative sepsis							**<0.0001**
Unknown	44	0.1	42	0.1	2	0.1	
None	68,367	88.6	65,081	88.9	3286	84	
SIRS	8157	10.6	7601	10.4	556	14.2	
Sepsis	550	0.7	489	0.7	61	1.6	
Septic shock	26	0	19	0	7	0.2	
ASA classification							**<0.0001**
1-No disturb	363	0.5	357	0.5	6	0.2	
2-Mild disturb	13,392	17.4	13,045	17.8	347	8.9	
3-Severe disturb	50,123	65	47,758	65.2	2365	60.5	
4-Life threat	13,266	17.2	12,072	16.5	1194	30.5	
Functional status							**<0.0001**
Unknown	645	0.8	608	0.8	37	0.9	
Independent	59,927	77.7	57,186	78.1	2741	70.1	
Partially dependent	14,138	18.3	13,166	18	972	24.8	
Totally dependent	2434	3.2	2272	3.1	162	4.1	
Admission to OR (d)							**<0.0001**
0 < 1	18,221	23.6	17,662	24.1	559	14.3	
1 < 2	41,281	53.5	39,771	54.3	1510	38.6	
2 < 3	12,151	15.8	11,294	15.4	857	21.9	
3 < 4	3151	4.1	2839	3.9	312	8	
4 < 5	1046	1.4	910	1.2	136	3.5	
5≤	1294	1.7	756	1	538	13.8	

ASA = American Society of Anesthesiologist, BMI = body mass index, CHF = congestive heart failure, COPD = chronic obstructive pulmonary disease, eLOS = extended length of hospital stay, Hx = history

Univariate analysis, using χ^2^ and Fisher exact tests.

Bold indicates *P*<0.05.

Preoperative variables were fitted into a forward, backward, and stepwise logistic regression (–C, respectively) during model development, and all three methods resulted in the same model (*P* = 0.3287) (Table 2). The model training data set, using two-thirds of the NSQIP population, had an AUC of 0.73, and the model validation data set, using the remaining one-third of the NSQIP population, had an AUC of 0.74. The Brier scores for both the training and validation data sets were small (0.027 and 0.027, respectively), indicating good model prediction.^16^

**Figure 2:**
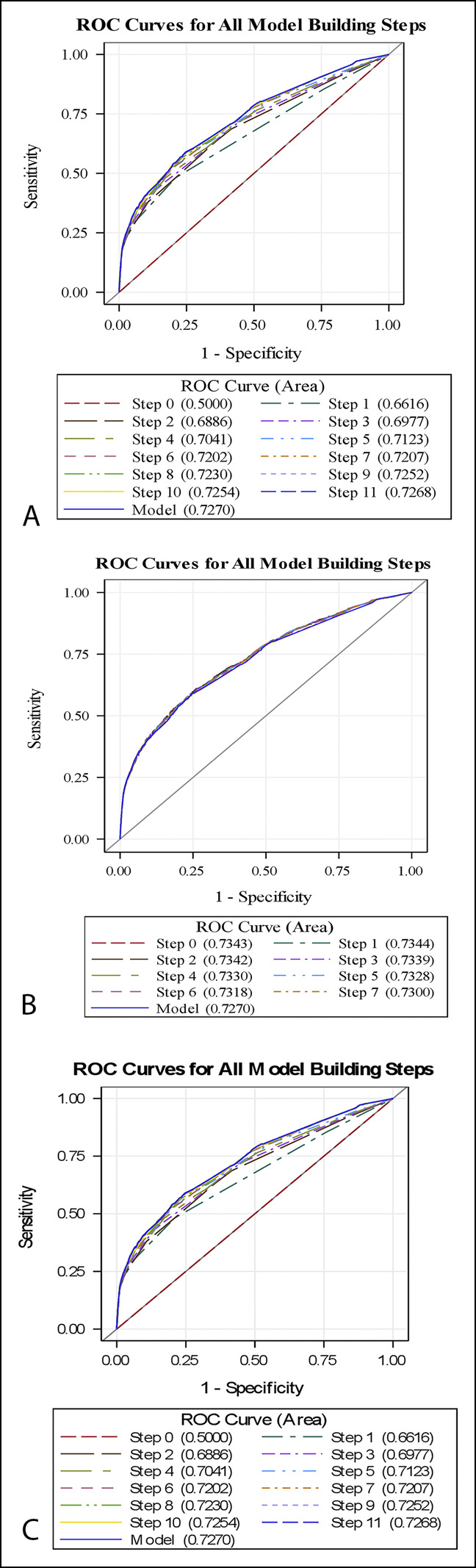
**A–C**, Preoperative variables were fitted into a forward, backward, and stepwise logistic regression (respectively) during model development, and all three methods resulted in the same model (*P* = 0.3287).

**Table 2:** Logistic Regression Analysis for Extended Length of Hospital Stay Using the National Surgical Quality Improvement Program Database

Variables	*P*	OR	95% CI	
Sex				
Female	Reference			
Male	**<0.0001**	1.568	1.385	1.774
Race				
White	Reference			
Black	**0.0084**	1.424	1.095	1.852
Other	**<0.0001**	2.043	1.574	2.651
Dyspnea				
None	Reference			
Yes	**<0.0001**	1.53	1.265	1.851
Ventilator				
No	Reference			
Yes	**<0.0001**	4.63	2.397	8.941
COPD				
No	Reference			
Yes	**0.0004**	1.358	1.145	1.609
Ascites				
No	Reference			
Yes	**0.0131**	2.854	1.246	6.538
CHF				
No	Reference			
Yes	**0.0094**	1.358	1.078	1.712
Renal failure				
No	Reference			
Yes	**0.0439**	1.7	1.015	2.85
Dialysis				
No	Reference			
Yes	**0.0179**	1.486	1.071	2.062
Systemic sepsis within 48 hours before surgery				
None	Reference			
SIRS	**<0.0001**	1.594	1.356	1.874
Sepsis	**0.0153**	1.881	1.129	3.135
Septic shock	0.5044	1.694	0.36	7.971
ASA classification				
1 and 2	Reference			
3 and 4	**<0.0001**	1.757	1.406	2.195
Admission to OR (d)				
<1	Reference			
1 < 2	0.3731	1.084	0.907	1.296
2 < 3	**<0.0001**	1.725	1.406	2.117
3 < 4	**<0.0001**	2.735	2.106	3.551
4 < 5	**<0.0001**	4.168	2.996	5.798
≥5	**<0.0001**	20.145	15.975	25.404

ASA = American Society of Anesthesiologist, CHF = congestive heart failure, CI, confidence interval, COPD = chronic obstructive pulmonary disease

Bold indicates *P*<0.05.

**Figure 3:**
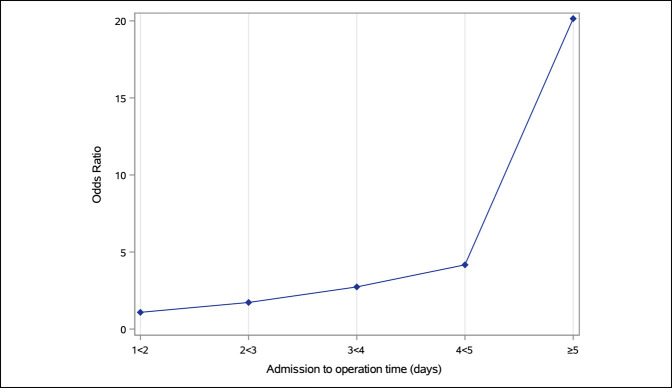
Line graph demonstrating the probability of having an extended length of hospital stay based on time from admission to operation (reference <1 day). Admission-to-operation time demonstrated a dose-response relationship with the probability of having an extended length of hospital stay.

Male sex, dyspnea, ventilator use, COPD, and ASA class 3 and 4 were associated with higher odds of having an eLOS. Increased admission-to-operation time correlated with eLOS, as shown in Figure 3.

A univariate analysis was used to compare postoperative complications between eLOS patients and non-eLOS patients, shown in Table 3. eLOS patients were more likely to have almost every postoperative complication, besides wound disruption. Multivariate logistic regression analysis demonstrated that patients with postoperative acute renal failure had the highest likelihood of eLOS (odds ratio [OR] 7.664), followed by ventilator use >48 hours (OR 4.784) and pneumonia (OR 4.332) (Table 4).

**Table 3:** Univariate Analysis for Postoperative Complications: eLOS Versus Non-eLOS Patients

Variable	Total		eLOS				*P*
	N	%	No		Yes		
			N	%	N	%	
Total patients	77,144	100	68,234	100	8910	100	
Superficial surgical site infection							**<0.0001**
No	76,689	99.4	67,867	99.5	8822	99	** **
Yes	455	0.6	367	0.5	88	1	** **
Deep incisional surgical site infection							**<0.0001**
No	76,955	99.8	68,084	99.8	8871	99.6	** **
Yes	189	0.2	150	0.2	39	0.4	** **
Organ space infection							**0.0004**
No	76,995	99.8	68,112	99.8	8883	99.7	** **
Yes	149	0.2	122	0.2	27	0.3	** **
Wound disruption							0.1299
No	77,083	99.9	68,188	99.9	8895	99.8	** **
Yes	61	0.1	46	0.1	15	0.2	** **
Pneumonia							**<0.0001**
No	74,813	97	66,851	98	7962	89.4	** **
Yes	2331	3	1383	2	948	10.6	** **
Unplanned intubation							**<0.0001**
No	76,632	99.3	67,988	99.6	8644	97	** **
Yes	512	0.7	246	0.4	266	3	** **
Pulmonary embolism							**<0.0001**
No	76,623	99.3	67,856	99.4	8767	98.4	** **
Yes	521	0.7	378	0.6	143	1.6	** **
Progressive renal insufficiency							**<0.0001**
No	76,905	99.7	68,081	99.8	8824	99	** **
Yes	239	0.3	153	0.2	86	1	** **
Acute renal failure							**<0.0001**
No complication	76,988	99.8	68,171	99.9	8817	99	** **
Acute renal failure	156	0.2	63	0.1	93	1	** **
Urinary tract infection							**<0.0001**
No	73,715	95.6	65,604	96.1	8111	91	** **
Yes	3429	4.4	2630	3.9	799	9	** **
Myocardial infarction							**<0.0001**
No	76,167	98.7	67,573	99	8594	96.5	** **
Yes	977	1.3	661	1	316	3.5	** **
Bleeding transfusions							**<0.0001**
No	57,080	74	51,047	74.8	6033	67.7	** **
Yes	20,064	26	17,187	25.2	2877	32.3	** **
DVT							**<0.0001**
No	76,330	98.9	67,599	99.1	8731	98	** **
Yes	814	1.1	635	0.9	179	2	** **
Sepsis							**<0.0001**
No	76,358	99	67,662	99.2	8696	97.6	** **
Yes	786	1	572	0.8	214	2.4	** **
Septic shock							**<0.0001**
No	76,858	99.6	68,052	99.7	8806	98.8	** **
Yes	286	0.4	182	0.3	104	1.2	** **
Stroke/CVA with neurologic deficit							**<0.0001**
No	76,736	99.5	67,986	99.6	8750	98.2	** **
Yes	408	0.5	248	0.4	160	1.8	** **
Return to OR							**<0.0001**
No	75,190	97.5	66,781	97.9	8409	94.4	** **
Yes	1951	2.5	1450	2.1	501	5.6	** **
Ventilator > 48 hr							**<0.0001**
No	76,865	99.6	68,160	99.9	8705	97.7	** **
Yes	279	0.4	74	0.1	205	2.3	** **

Deep Vein Thrombosis and Cerebrovascular Accident DVT = deep vein thrombosis, CVA = cerebrovascular accident eLOS, extended length of hospital stay, OR = odds ratio

Bold indicates *P*<0.05.

**Table 4:** Multivariate Logistic Regression for Postoperative Complications Associated With Extended Length of Hospital Stay in the National Surgical Quality Improvement Program Population

Variable	*P*	OR	95% CI	
Superficial incisional infection	**0.002**	1.629	1.196	2.22
Deep incisional infection	0.9904	0.997	0.617	1.612
Organ/space infection	0.6259	0.872	0.502	1.513
Pneumonia	**<0.0001**	4.351	3.88	4.881
Unplanned intubation	**0.0001**	1.803	1.338	2.43
Pulmonary embolism	**<0.0001**	1.891	1.428	2.503
Progressive renal insufficiency	**<0.0001**	2.339	1.629	3.359
Acute renal failure	**<0.0001**	7.782	5.423	11.169
Urinary tract infection	**<0.0001**	2.538	2.268	2.84
Myocardial infarction	**<0.0001**	2.289	1.898	2.762
Blood transfusion	**<0.0001**	1.247	1.16	1.34
DVT	**0.0003**	1.561	1.227	1.985
Sepsis	**0.0002**	1.498	1.207	1.859
Septic shock	0.0617	0.704	0.488	1.017
Stroke	**<0.0001**	3.573	2.747	4.648
Return to the operating room	**<0.0001**	2.48	2.13	2.888
On ventilator greater than 48 hours	**<0.0001**	3.701	2.559	5.352

CI = confidence interval, OR = odds ratio

Bold indicates *P*<0.05.

## Discussion

In our study of 77,144 geriatric hip fracture patients, eLOS patients differed from non-eLOS patients by several demographic and comorbid traits. Male sex, non-White ethnicity, pulmonary complications, and ASA class were all significant risk factors for eLOS. These results corroborate the findings of other length of stay studies. Gil et al^5^ reported that patients with the longest length of stay in the trauma population were more likely to be male, African American, and have preexisting solid organ comorbidities. Garcia et al,^9^ in a retrospective review of 660 geriatric hip fracture patients, found that male sex and an increasing ASA score were significant risk factors for an increased LOS, using multivariate analysis. Although these traits are not necessarily modifiable, they are easily identified. The early identification of nonmodifiable factors that are associated with eLOS may assist in cost projections and managing patient and provider expectations.

Patients with an increased admission-to-operation time, even after controlling for comorbidities, were more likely to have an eLOS, and the risk was dose dependent; the longer the wait until surgery, the greater the risk of eLOS. This finding is in agreement with Pincus et al,^13^ who performed a Canadian population-based study of 42,230 adult hip fracture patients. They found that overall cost of care was significantly lowered when hip fracture surgery was performed within 1 day of admission. Alvi et al,^14^ in a review of geriatric hip fractures from 2011 to 2012, performed a comorbidity-adjusted analysis showing no time-to-surgery–related difference in postoperative complications (*P* = 0.143), but an increase in the total LOS (*P* < 0.001) and surgery-to-discharge time (*P* < 0.001) in patients whose admission-to-operation time was >48 hours. They stated that the timeliness of surgical intervention in this population causes no effect on overall complications, readmissions, or 30-day mortality, but an admission-to-operation time of >48 hours is associated with costly increases in LOS. Hecht et al^15^ developed a predictive model for LOS in geriatric hip fracture patients and found that for each day surgery was delayed, LOS increased by 0.75 days. The totality of the results of from our study and aforementioned studies suggest that hospitals and provider teams continue to investigate possible interventions to mitigate unnecessary delays to surgery.

The postoperative analysis in our study revealed that patients were at greatest risk of eLOS if they were on a ventilator >48 hours or developed pneumonia or acute renal failure. These results support the findings of previous studies. O'Keefe et al^2^ found pneumonia and acute renal failure to be two of the six complications that led to the greatest difference between observed and expected hospital admission costs in trauma patients. These results, along with our findings, suggest that patients who end up having an eLOS may not have been destined to do so at the time of hospital admission.^6^ In addition, inpatient complications, specifically pulmonary and renal complications, may serve as modifiable risk factors and targets for preemptive prevention. Bohl et al,^16^ in a retrospective review of 29,377 geriatric hip fracture patients, showed that significant risk factors for pneumonia included male sex, age >90, history of COPD, and body mass index <18.5 kg/m^2^. They recommended evidence-based interventions such as upright eating, incentive spirometry, elevating the head of the bed >30°, encouraging ambulation, and instituting a twice-daily oral hygiene practice with chlorhexidine. They also suggested the implementation of a standardized pneumonia prevention program including a pneumonia-specific nurse education, pneumonia prevention nursing checklist, and a pneumonia prevention order-set for physicians.^17^ These interventions were based on the work of Kazaure et al,^17^ who demonstrated a 43.6% decrease in postoperative pneumonia rate in surgical patients over a 5-year period after implementation of aforementioned measures. A similar percentage of decrease in pneumonia occurrence at ACS-NSQIP hospitals would represent approximately 6118 prevented pneumonia cases and a cost savings of more than $280 million. With regard to postoperative renal complications, Bennet et al^18^ investigated the incidence, risk factors, and outcomes of acute renal dysfunction in patients with a hip fracture. Risk factors included male sex, diabetes mellitus, vascular disease, hypertension, chronic renal disease, and use of ACE inhibitors, angiotensin-II receptor blockers or diuretics before their admission; cited strategies for prevention were optimization of preoperative volume status and oxygen delivery, and consideration of the risks and benefits of continuing nephrotoxic home medications. Brauner Christensen et al,^19^ in a retrospective cohort study, investigated the predictors of AKI after geriatric hip fracture. Patient with a postoperative AKI were more likely to be older and have preexisting heart disease. Modifiable risk factors included postoperative blood transfusion, corroborating prior studies in nonorthopaedic specialties, suggesting the avoidance of liberal transfusion practices.^20,21^ Shin et al found association between postoperative serum albumin levels and the development of AKI after hip fracture surgery in older adults. They propose that monitoring serum albumin levels may identify early postoperative hypoalbuminemia and aid in the early detection and prevention of AKI.

Several bundled payment models have recently replaced prior fee-for-service reimbursements to more adequately represent the economic burden conferred by the costliest patients. Yet, despite its goal of reducing healthcare expenditures, bundling payments for geriatric hip fracture patients without the proper risk adjustment may have egregious consequences, including disincentivizing hospitals and physicians from taking care of the most comorbid patients.^22^ Our results suggest that consideration of certain factors such as sex, ethnicity, and pulmonary and renal comorbidities in bundles may be beneficial and improve equitability among providers. Of the total costs associated with geriatric hip fracture care, between 44% and 57% can be attributed to the inpatient stay alone, with LOS and inpatient complications being the strongest drivers of expenditures.^23,24^ Care providers' efforts to address modifiable risk factors that lead to eLOS could diminish costs. However, geriatric hip fracture providers do not have ample time to preoperatively optimize this nonelective surgical patient population, and thus a thorough understanding of prehospital risk factors for eLOS is paramount in a bundled payment system. With this understanding, bundled costs can be appropriately calibrated and equitable reimbursements can be better ensured. By taking into account our model, care facilities who regularly take care of complex geriatric hip fracture patients could be at less of a financial disadvantage. Ultimately, this may guard against disincentivizing hospitals from accepting sicker patients.

Understanding our results in the context of this study's limitations is critical. First, the study was limited by shortcomings common to all database studies. The design of this study was retrospective and excluded patients with missing or incomplete data. Second, although LOS is a significant driver of hospitalization cost, it is not equivalent to hospitalization cost or resource utilization proper. Excessive cost and resource utilization typically involve a degree of discretionary overuse and can occur via multiple domains not mentioned in this study, emergency department visits, prescription medications, outpatient and inpatient therapy services, and placement in extended care facilities, for example. The valuable inclusion of domains such as these could be only be performed with more granular data and surveillance further from the 30-day postoperative reporting range of the NSQIP database. Furthermore, we acknowledge that in this population of patients, there exist causes for eLOS that are nondiscretionary; some complications are inevitable. However, we feel that the risk factors identified in this current study that are associated with patients with the lengthiest inpatient stays still provide valuable information. These risk factors are worthy targets of intervention to mitigate the catastrophic inpatient complications that can be prevented. Third, we excluded patients who died in the hospital from our analysis. Including them may have biased our results, as some patients may have been destined for eLOS but did not live long enough to qualify. Of note, this situation was a rare, occurring in only 5.5% of the total patient population. Finally, we recognize that admission-to-surgery time biases overall hospital LOS and thus the risk one has of having an eLOS.

This study identified risk factors associated with an eLOS after a hip fracture in the geriatric population. In addition, a model predictive of having an eLOS in this population was built and validated. Despite its limitations, this study adds valuable data to the literature regarding the factors that influence eLOS after geriatric hip fracture. To our knowledge, this study is the first to characterize the geriatric hip fracture patients with the longest LOS as their own subgroup. Furthermore, we created and validated a statistical model predictive of eLOS with universally collected variables, which may aid in the creation of more accurate reimbursement models that best reflect the cost, length, and complexity of an inpatient hospital stay after a geriatric hip fracture. Future studies are needed to investigate possible preoperative and postoperative interventions to prevent patients from reaching eLOS status.

## References

[1] DiMaggio C Ayoung-Chee P Shinseki M: Traumatic injury in the United States: In-patient epidemiology 2000-2011. Injury 2016;47:1393-1403. 27157986 10.1016/j.injury.2016.04.002 PMC5269564

[2] O'Keefe GE Maier RV Diehr P Grossman D Jurkovich GJ Conrad D: The complications of trauma and their associated costs in a level I trauma center. Arch Surg 1997;132:920-924. 9267280 10.1001/archsurg.1997.01430320122021

[3] Cohen SB: The concentration of health care expenditures and related expenses for costly medical conditions, 2012, in Statistical Brief (Medical Expenditure Panel Survey (US)). Rockville, MD, 2001. NBK470837 [bookaccession]. 29281226

[4] Mitchell EM Machlin SR, Concentration of health expenditures and selected characteristics of high spenders, 2015, in Statistical Brief (Medical Expenditure Panel Survey (Us)). Rockville, MD, U.S. civilian noninstitutionalized population, 2001. NBK470846 [bookaccession]. 29281224

[5] Gil LA Kothari AN Brownlee SA: Superusers: Drivers of health care resource utilization in the national trauma population. Surgery 2018;164:848-855. 30093276 10.1016/j.surg.2018.04.046

[6] Lagoe RJ Johnson PE Murphy MP: Inpatient hospital complications and lengths of stay: A short report. BMC Res Notes 2011;4:135. 21545741 10.1186/1756-0500-4-135 PMC3098808

[7] Roberts KC Brox WT Jevsevar DS Sevarino K: Management of hip fractures in the elderly. J Am Acad Orthop Surg 2015;23:131-137. 25624365 10.5435/JAAOS-D-14-00432

[8] Miyamoto RG Kaplan KM Levine BR Egol KA Zuckerman JD: Surgical management of hip fractures: An evidence-based review of the literature. I: Femoral neck fractures. J Am Acad Orthop Surg 2008;16:596-607. 18832603 10.5435/00124635-200810000-00005

[9] Garcia AE Bonnaig JV Yoneda ZT: Patient variables which may predict length of stay and hospital costs in elderly patients with hip fracture. J Orthop Trauma 2012;26:620-623. 22832431 10.1097/BOT.0b013e3182695416

[10] Burge R Dawson-Hughes B Solomon DH Wong JB King A Tosteson A: Incidence and economic burden of osteoporosis-related fractures in the United States, 2005-2025. J Bone Miner Res 2007;22:465-475. 17144789 10.1359/jbmr.061113

[11] Lott A Haglin J Belayneh R Konda SR Egol KA: Admitting service affects cost and length of stay of hip fracture patients. Geriatr Orthop Surg Rehabil 2018;9:2151459318808845. 30479850 10.1177/2151459318808845 PMC6249656

[12] Konda SR Johnson JR Kelly EA Chan J Lyon T Egol KA: Can we accurately predict which geriatric and middle-aged hip fracture patients will experience a delay to surgery? Geriatr Orthop Surg Rehabil 2020;11:2151459320946021. 32821470 10.1177/2151459320946021 PMC7412893

[13] Pincus D Wasserstein D Ravi B: Medical costs of delayed hip fracture surgery. J Bone Joint Surg Am 2018;100:1387-1396. 30106820 10.2106/JBJS.17.01147

[14] Alvi HM Thompson RM Krishnan V Kwasny MJ Beal MD Manning DW: Time-to-surgery for definitive fixation of hip fractures: A look at outcomes based upon delay. Am J Orthop (Belle Mead NJ) 2018;47, doi: 10.12788/ajo.2018.007110.12788/ajo.2018.0071. 30296323

[15] Hecht G Slee CA Goodell PB Taylor SL Wolinsky PR: Predictive modeling for geriatric hip fracture patients: Early surgery and delirium have the largest influence on length of stay. J Am Acad Orthop Surg 2019;27:e293-e300. 30358636 10.5435/JAAOS-D-17-00447 PMC6411423

[16] Bohl DD Sershon RA Saltzman BM Darrith B Della Valle CJ: Incidence, risk factors, and clinical implications of pneumonia after surgery for geriatric hip fracture. J Arthroplasty 2018;33:1552-1556.e1. 29289445 10.1016/j.arth.2017.11.068

[17] Kazaure HS Martin M Yoon JK Wren SM: Long-term results of a postoperative pneumonia prevention program for the inpatient surgical ward. JAMA Surg 2014;149:914-918. 25054486 10.1001/jamasurg.2014.1216

[18] Bennet SJ Berry OM Goddard J Keating JF: Acute renal dysfunction following hip fracture. Injury 2010;41:335-338. 19729159 10.1016/j.injury.2009.07.009

[19] Brauner Christensen J Aasbrenn M Sandoval Castillo L: Predictors of acute kidney injury after hip fracture in older adults. Geriatr Orthop Surg Rehabil 2020;11:2151459320920088. 32313715 10.1177/2151459320920088 PMC7160769

[20] Freeland K Hamidian Jahromi A Duvall LM Mancini MC: Postoperative blood transfusion is an independent predictor of acute kidney injury in cardiac surgery patients. J Nephropathol 2015;4:121-126. 26457259 10.12860/jnp.2015.23 PMC4596296

[21] Karrowni W Vora AN Dai D Wojdyla D Dakik H Rao SV: Blood transfusion and the risk of acute kidney injury among patients with acute coronary syndrome undergoing percutaneous coronary intervention. Circ Cardiovasc Interv 2016;9:e003279. 27582110 10.1161/CIRCINTERVENTIONS.115.003279

[22] Konda SR Lott A Egol KA: Development of a value-based algorithm for inpatient triage of elderly hip fracture patients. J Am Acad Orthop Surg 2020;28:e566-e572. 31567901 10.5435/JAAOS-D-18-00400

[23] Malik AT Khan SN Ly TV Phieffer L Quatman CE: The “hip fracture” bundle-experiences, challenges, and opportunities. Geriatr Orthop Surg Rehabil 2020;11:2151459320910846. 32181049 10.1177/2151459320910846 PMC7059231

[24] Kates SL Blake D Bingham KW Kates OS Mendelson DA Friedman SM: Comparison of an organized geriatric fracture program to United States government data. Geriatr Orthop Surg Rehabil 2010;1:15-21. 23569657 10.1177/2151458510382231 PMC3597292

